# A comparison of inversion test holding methods for broilers

**DOI:** 10.1016/j.psj.2024.104163

**Published:** 2024-08-02

**Authors:** A. Perretti, S. Weimer

**Affiliations:** Department of Poultry Science, University of Arkansas, Fayetteville, AR 72701, USA

**Keywords:** broiler, behavior, inversion test, fear, method

## Abstract

Objective, robust, and repeatable assessments of fear responses of poultry can lead to improvements in research techniques, the validity of test results, and ultimately bird welfare. The objective of this study was to examine the effect of 2 different holding methods on broiler fear responses during the inversion test, a standardized method of assessing fear in poultry. On D15 and D34 mixed-sex broilers (N = 80) were inverted and held either by their shanks (N = 40) or feet (N = 40) at arm's length away from the experimenter for 30 s. The frequency of wing flapping, head movement, attempts to right, and vocalizations were observed from video recordings collected during the inversion test. Vocalizations at D15 and D34 were not different when comparing holding methods. Frequency of attempts to right and wing flapping at D15 were greater (*P* < 0.001) for birds held by their feet (0.90 and 16.6, respectively) than when they were held by their shanks (0.20 and 0.73, respectively). Similarly, on D34 wing flapping remained greater (*P* < 0.001) for birds held by their feet (23.8) compared to their shanks (8.1). Conversely, head movements were greater (*P* = 0.05) for birds held by their shanks (9.2) compared to their feet (6.3) on D15. Within both the shank and feet holding method, vocalizations at D15 were greater (30.7 and 35.6, respectively) compared to D34 (11.93 and 15.38, respectively) (*P* < 0.001). There was no holding method effect on head movements within each age. These results suggest that behaviors observed during the inversion test can be influenced by the inversion holding method and that handling should be standardized while conducting behavioral tests to assess fear in poultry.

## INTRODUCTION

Fear is a reaction to an environmental stimulus that is perceived as a threat. An animal's response to that stimulus may be freezing, attempts to escape, or elevated heart rates (Broom, 1991), but the animal's ability to cope with the exposure to the stimulus is critical in making decisions and avoiding potential dangers (Broom, 1991; Boissy, 1995). Fear responses can be measured physiologically (e.g., stress hormone levels) or through behavioral observations (e.g., responsiveness, avoidance). Observations of an animal's behavior in stressful events can be used by producers to adjust their management strategies to optimize and improve the animal's welfare. Researchers use fear tests to recreate known stressors that an animal may experience in a controlled and systematic setting. The tonic immobility test, novel arena test, human approach test, and inversion test are some examples of objective, commonly used fear tests that help researchers gain a better understanding of the fear response in animals (Gray, 1987; Forkman et al., 2007).

Broilers can experience stressors during any phase of production. One source of stress is catching for transport to slaughter. Duncan et al. (1986) found that broilers caught manually had longer durations of tonic immobility and elevated heart rates compared to those mechanically caught. During manual catching on commercial broiler farms, broilers are picked up, inverted by their legs, and carried while inverted, to transport modules or crates. The inversion test was developed by Newberry and Blair (1993) to experimentally recreate the bird's experience of human handling during manual catching. The behavioral responses from the Inversion Test are used as a guide for researchers to assess fear and stress. During the Inversion Test, the handler picks up the test bird from their pen and inverts the bird while holding onto their legs (Newberry and Blair, 1993). Video recordings of behavioral tests can be used for more in-depth behavioral analysis and behaviors include vocalizations, wing flapping, attempts to right, and head movements. Previous studies used the Inversion Test to measure the effect of diet (Newberry and Blair, 1993), photoperiod (Archer and Mench, 2014), lighting (House et al., 2020), and inter-species socialization (Schanz et al., 2022) on the fear response of broilers.

The objective of this study was to examine the effects of 2 holding methods (shank and feet) on broiler behavior during the inversion test at 2 ages (D15 and D34). We hypothesized that broilers held by their feet would have a greater frequency of attempts to right and wing flapping because birds were less restrained compared to those held by their shanks and that the frequency of behaviors would decrease with age.

## MATERIALS AND METHODS

### Animals and Housing

The study was conducted at the University of Arkansas Poultry Research Farm and all methods and procedures were approved by the University of Arkansas Institutional Animal Care and Use Committee. Day-of-hatch Cobb MX x Cobb 500 broiler chicks (N = 274) were randomly assigned across 16 pens (1.5m x 3m) averaging 17 chicks per pen. The birds were randomly assigned a holding method at D15. Housing conditions were maintained according to the Cobb-Vantress broiler management guide (Cobb-Vantress, 2021).

### Inversion Test

**Figure 1:**
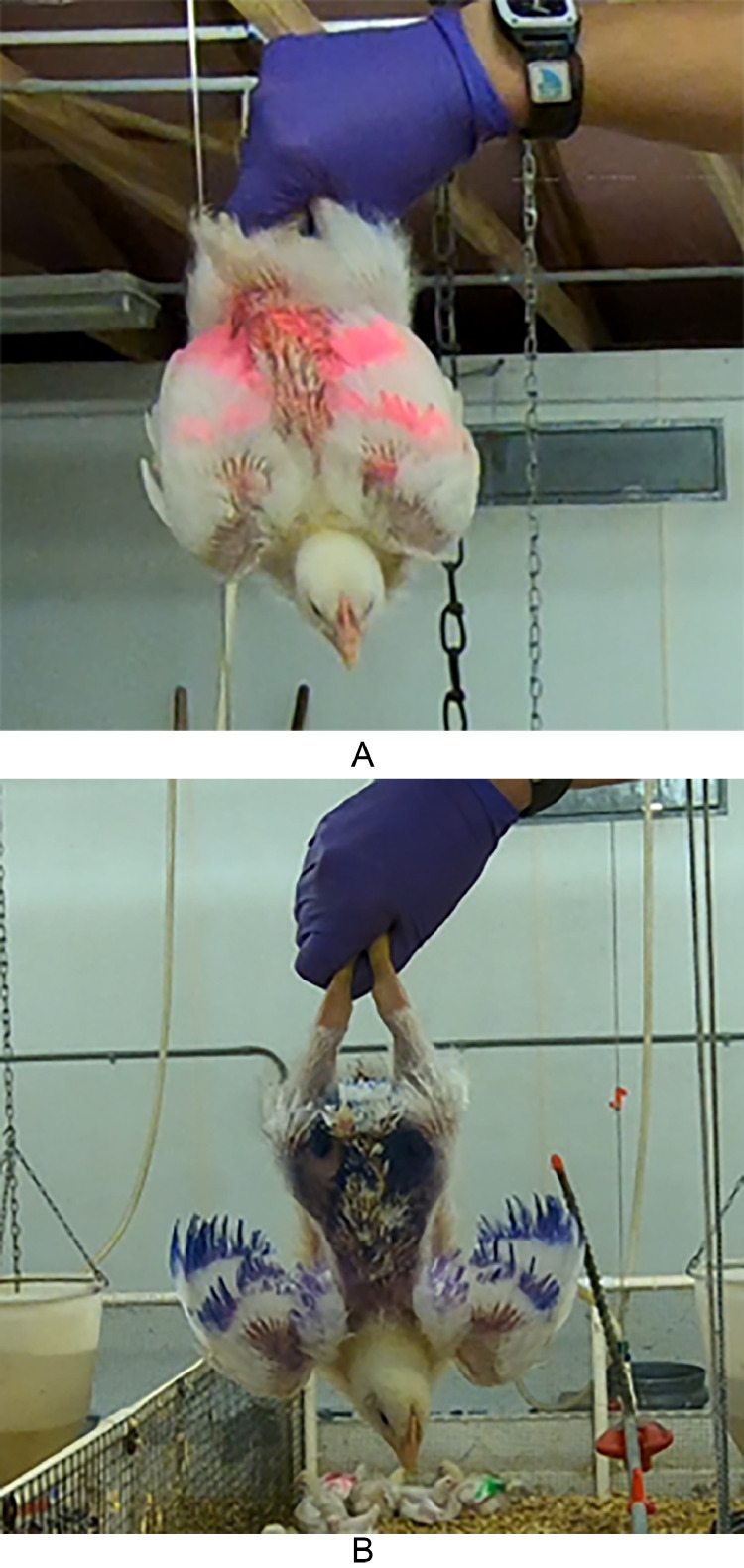
Images of 2 different holding methods during the inversion test where individual birds were inverted and held either by their (A) shanks or (B) feet.

The Inversion Test was conducted on 5 birds/pen (N = 80) randomly selected on D15. At D15, the birds were uniquely color-marked with agricultural spray for identification and testing on D34. The Inversion Test followed the procedures described in Newberry and Blair (1993) with modifications to the holding method. At the start of the test, the handler stepped into each pen, picked up 1 bird by its body with both hands, transitioned either both shanks (shanks holding method) or both feet (feet holding method) into 1 hand, lifted and inverted the bird up at arm's length away at shoulder height for 30 s (Figure 1). Birds were held by their shanks in half of the pens (N = 40) and by their feet in the other half (N = 40). Due to the high variability of behavior data, the same birds tested on D15 were also tested on D34, except for 2 mortalities between testing days, in which the birds were replaced with 2 birds from the same pens. Upon inversion, the recorder outside of the pen recorded the time of day and notified the handler when 30 s had elapsed. Once the test ended, the bird was lowered, the handler placed their free hand under the bird's chest to bring the bird back to an upright position and placed the bird onto their feet in the pen. Video was recorded using a GoPro camera (GoPro Hero 8, San Mateo, CA) mounted atop a tripod located in the walkway next to the recorder. The number of vocalizations, head movements, attempts to right, and wing flapping for each trial were counted for each bird from video recordings. Vocalizations were defined as any sound emanating from the bird (Grandin, 1998). Wing flapping was defined as simultaneous up and down movement of both wings (adapted from Vas et al., 2023). An attempt to right was defined as the bird twisting and lifting their body in an effort to regain an upright position, typically bending at a 90° angle. Head movement was defined as the bird actively moving their head and neck in any direction. Some behaviors occurred simultaneously with others (e.g., vocalizing while wing flapping, wing flapping while righting) and in these cases, both behaviors were counted and coded separately.

### Statistical Analysis

All statistical analyses of data were conducted using JMP Pro 17 (SAS Institute Inc. Cary, NC). Comparison of AIC values were used to determine that data best fit normal distribution analysis. A 2-way ANOVA of the main effects of age, method, and their interactions, revealed that the interaction of age and holding method was not significant. Separate ANOVAs that included the random effect of bird ID nested within pen were used to compare the fixed effects of holding method (shanks and feet) within age and age (D15 and D34) within holding method on the count frequencies of each behavior. Significance was declared at *P* ≤ 0.05 and a trend at *P* ≤ 0.10. Descriptive statistics are reported for the proportion of birds that exhibited each behavior. The proportion of birds that exhibited each behavior were calculated by taking the total number of birds tested divided by the total number of birds that performed that behavior.

## RESULTS AND DISCUSSION

Newberry and Blair (1993) reported that handlers influenced Inversion Test Results on broiler body curling (attempts to right) and frequency of wing flaps and called for further research to identify handler features that can affect chicken flight and fear responses. The objective of this study was to examine if holding broilers either by their shanks or feet affected their behavior during the Inversion test at 2 ages. Results are reported in Table 1. There was no effect of holding method on the frequency of vocalizations at D15 (*P* = 0.15) or D34 (*P* = 0.11). The holding method may not influence number of vocalizations, likely because this behavior is not as dependent on the physical ability of the birds as the other observed behaviors were. When birds were inverted by their feet at D15, they exhibited greater (*P* < 0.0001) frequencies of attempts to right (0.9) and wing flapping (16.6) compared to inversion by their shanks (0.2 and 0.73, respectively). Holding birds with their feet increased opportunities for full-body flexibility in their movements, as birds held by their shanks received greater pressure and restraint on their hock joints. Conversely, D15 head movements were greater (*P* = 0.05) for birds inverted by their shanks (9.2) than by their feet (6.3). Head movements may have been greater when birds were held by their shanks because they had lower frequencies of wing flapping and righting. Like D15, birds held by their feet during inversion had a greater (*P* < 0.001) frequency of wing flaps (23.8) and a tendency (*P* = 0.06) for attempts to right to be greater (0.9) on D34 compared to their shanks (8.1 wing flaps and 0.4 attempts to right). The present results show that hand placement on broilers during the Inversion Test can affect the behavioral measures collected.

**Table 1:** Mean (± SE) number of wing flapping, righting, head movement, and vocalizations during the Inversion Test (30 s) using 2 holding methods (shanks and feet) at 2 ages (D15 and D34).

		Age		
Behavior	Method	D15	D34	*P*-value
Attempts to right	Shanks	0.20^b^ ± 0.08	0.38 ± 0.08	0.109
	Feet	0.90^a^ ± 0.21	0.90 ± 0.21	1.000
	*P*-value	<0.0001	0.0568	
				
Wing flaps	Shanks	0.73^b^^,^^z^ ± 1.09	8.10^b^^,^^y^ ± 1.09	<0.0001
	Feet	16.55^a^^,^^z^ ± 2.91	23.78^a^^,^^y^ ± 2.91	0.0109
	*P*-value	< 0.0001	<0.0001	
				
Head movements	Shanks	9.18^a^^,^^y^ ± 0.95	4.98^z^ ± 0.95	0.0002
	Feet	6.28^b^^,^^y^ ± 0.67	4.55^z^ ± 0.67	0.048
	*P*-value	0.046	0.592	
				
Vocalizations	Shanks	30.7^y^ ± 1.98	11.93^z^ ± 1.98	<0.0001
	Feet	35.6^y^ ± 2.01	15.38^z^ ± 2.01	<0.0001
	*P*-value	0.157	0.107	

ab Means not sharing the same letter within each column indicates a significant difference between methods (Shanks vs. Feet) within each age for each behavior.

yz Means not sharing the same letter across each row indicates a significant difference between ages (D15 vs. 34) within each behavior.

Independent of holding method, all behaviors except frequency of attempts to right were greater (*P* < 0.003) at D15 than D34; younger birds exhibited 19.4 more vocalizations, 7.5 more wing flaps, and 1.97 more head movements than older birds (data not shown). As broilers age, their ability to perform physically strenuous behaviors decreases. Newberry and Blair (1993) found that older birds had greater wing flapping durations than younger birds (5.3 s and 3.3 s, respectively) during the Inversion Test. This same trend was found in the current study with birds at D15 that had an average duration of 2.7 s for wing flapping bouts, while D34 birds had an average duration of 3.3 s for wing flapping bouts (data not shown). Newberry and Blair (1993) reported that older birds (6 wk) wing flapped at a slower rate because the larger wings of older birds took more time to flap than smaller wings of younger birds, thus a longer duration of wing flapping. In addition to inverting the test bird for 30 s, Newberry and Blair (1993) carried the inverted bird for 60 s directly after the initial inversion. They found 96% of 3-wk-old broilers vocalized compared to 82% of 6-wk-old birds while they were inverted and carried. In the current study, 100% of D15 birds and 96% of D34 birds vocalized, 39% of D15 birds and 70% of D34 birds wing flapped, 33% of D15 birds and 36% of D34 birds attempted to right, and 77% of D15 birds and 75% of D34 birds moved their heads. Younger birds may vocalize more than older birds because of the gravity of the additional weight and the bird's lack of a diaphragm, which could have led to difficulty breathing to vocalize during inversion (Newberry and Blair, 1993).

Before the test bird is inverted for the Inversion Test, the bird is picked up by the handler, which is a circumstance that could add handling stress. Test location is another component that could add stress variability to the Inversion Test. Schanz et al. (2022) conducted the Inversion Test in a test arena, while Newberry and Blair (1993) and the current study conducted the test in the home pen. There may be some elements in the test environment that could impact behaviors in both a test arena (e.g., novel environment and isolation) and in the home pen (e.g., conspecific visibility and vocalizations). In Newberry and Blair (1993), broilers that had greater frequencies of wing flapping and body curling (attempts to right) during the Inversion Test also had longer latencies and fewer attempts required to induce tonic immobility in the Tonic Immobility Test. In addition to the impact of environmental characteristics, age-related body weight could affect the bird's physical ability to perform a behavior. Body weight was not recorded in the current study and may have influenced behavior; however, it was noted that the physical fatigue of 1 handler inverting the heavier broilers at D34 resulted in a second handler completing the Inversion Tests for the remaining birds. The body weight and number of birds, time, and physical capabilities of researchers, and weight should be considered when conducting the Inversion Test on poultry. One potential solution to handler fatigue would be the use of a device or stationary metal shackles that restrain the bird during inversion.

The results from the current study indicate that hand placement on the legs of broilers while conducting the Inversion Test can affect test results as wing flapping, attempts to right, vocalizations, and head movements were affected by the inversion holding method. Holding birds by their shanks is more restraining than holding them by their feet because the hand of the handler grips a greater surface area of the bird's legs that is proximal to the feet, and thus creates more resistance when the bird attempts to right themselves. The results of the current study suggest there is importance in standardizing holding methodologies when conducting the inversion test. Additionally, many aspects of a fear test can be impacted by procedures and if not standardized may impact the accuracy and validity of test results.

## DISCLOSURES

The authors declare no conflicts of interest.
